# Unveiling the Hidden Cause: A Case of Persistent Dysphagia

**DOI:** 10.7759/cureus.70990

**Published:** 2024-10-07

**Authors:** Rachel V Lee, Dupinder Singh, Gregory E Idos

**Affiliations:** 1 Department of Internal Medicine, City of Hope Comprehensive Cancer Center, Duarte, USA

**Keywords:** hardware malufunction, neurosurgery, rare cause of dysphagia, renal cell carcinoma metastasis, traumatic cervical spine injury

## Abstract

Esophageal impingement due to hardware failure is a rare complication of cervical spine fusion surgery; dysphagia, on the other hand, is a much more common complication. Here, we present the case of a 72-year-old man with a rare complication of anterior cervical discectomy and fusion (ACDF) induced by a migrated screw, causing dysphagia. The patient, with a history of metastatic renal cell carcinoma, had been undergoing treatment with cabozantinib and nivolumab for a year when he developed new-onset dysphagia over several months. Subsequent investigations, including contrast-enhanced cervical spine CT and esophagogastroduodenoscopy, revealed a prior C5-C7 anterior spinal fusion, with a C7 vertebral body screw protruding into the esophagus. The patient had been in an automobile accident in 2020 but reported no complications until the onset of dysphagia nearly two years later. A modified barium swallow test/fluoroscopic esophagogram confirmed that the protruding screw was responsible for the dysphagia. Afterward, he underwent successful endoscopic surgery to remove the screw, with complete resolution of his dysphagia and no postoperative complications. This case underscores the importance of prompt identification and intervention in managing screw migration into the esophagus, with multidisciplinary collaboration playing a crucial role in achieving successful outcomes.

## Introduction

Anterior cervical discectomy and fusion (ACDF) is a surgical procedure used to remove herniated or degenerative discs from the neck. This surgery is considered safe and is one of the most common cervical spine surgeries performed in the US, with an estimated 137,000 ACDF procedures performed per year [1]. The morbidity rate of ACDF ranges from 13.2% to 19.3% [1]. Complications can include dysphagia (1.7-9.5%), postoperative hematoma (0.4-5.6%), and esophageal perforation (0.3-0.9%) [1]. Though rare, these complications can occur, such as in this patient's case. Hardware failure, particularly instrument backout, is also possible, accounting for only approximately 0.1% of the reported complications [2]. This backout and movement can penetrate the surrounding structures, such as the esophagus, leading to symptoms of dysphagia or odynophagia. Patients with low bone density are at a higher risk of screw migration. Even patients with normal bone density but a history of multiple spine surgeries may have weak structural stability, increasing the likelihood of screw pullout and migration [2]. This migration can be dangerous and fatal. In one retrospective study with 1015 patients who underwent first-time ACDF for cervical radiculopathy and/or myelopathy for degenerative disc disease, esophageal perforation occurred in 0.3% of patients. The mortality rate was 0.1%, with the death occurring secondary to an esophageal perforation [3].

This case discusses the rare and possibly fatal complication of a migrated screw causing dysphagia in a patient who underwent ACDF surgery. In other reported cases of screw migration, patients have experienced symptoms such as neck pain or hoarseness; however, dysphagia was the only symptom observed in our case. Symptomatic delayed screw migration (occurring after over 10 years) is also less common, and the timing makes this case especially unique [4].

## Case presentation

**Figure 1:**
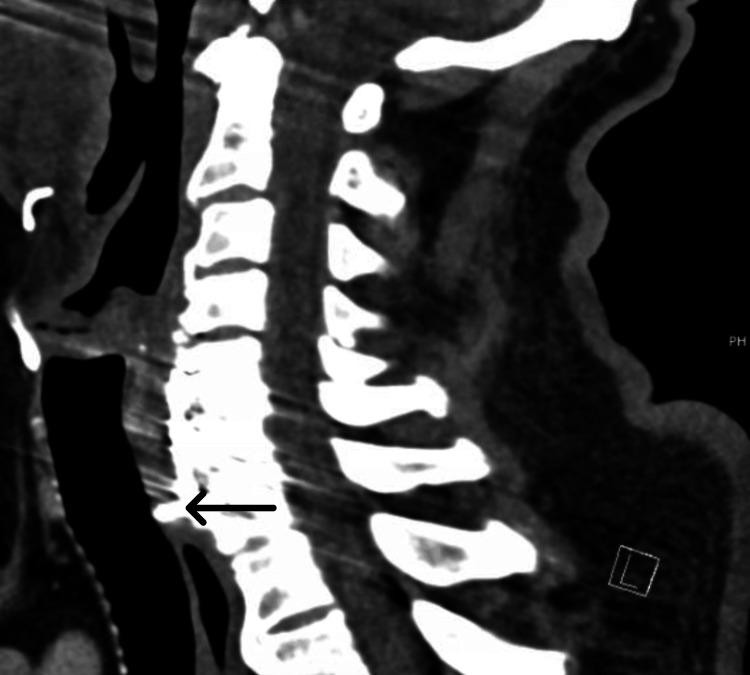
Computed tomography scan of the cervical spine with IV contrast reveals a C5-C7 anterior spinal fusion with a C7 screw protruding into the esophagus.

**Figure 2:**
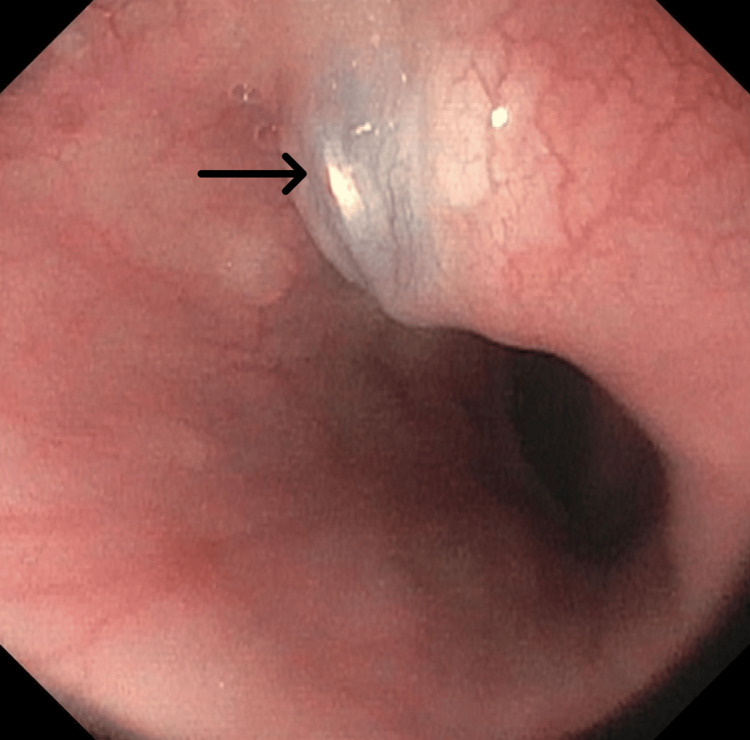
Esophagogastroduodenoscopy image showing a loose screw impinging on the patient’s esophagus.

A 72-year-old man with a history of type 2 diabetes mellitus, hyperlipidemia, chronic gout, anterior cervical discectomy in 2007, and automobile accident in 2020 was treated for metastatic renal cell carcinoma after a partial nephrectomy in 2016. This was complicated by a mass in the right chest that caused sternal destruction and chronic hematuria. The patient had been receiving a regimen of cabozantinib and nivolumab for one year. During this period, he complained of episodes of dysphagia and choking on solid foods over the previous few months. Two months prior to presentation, he choked on meat but was able to dislodge it by vomiting. Since then, he has been ensuring that he chewed his food thoroughly and replaced it with liquid-based calories. He did not experience dysphagia when consuming liquids and stated that he had undergone an upper endoscopy several years before but could not recall the results. Computed tomography (CT) (Figure 1) and esophagogastroduodenoscopy (Figure 2) were performed to determine the cause of dysphagia.

The initial differential diagnoses included metastasis of renal cell carcinoma, stricture, esophageal webs or rings, and Zenker’s diverticulum. However, contrast-enhanced cervical spine CT revealed that the patient had previously undergone C5-C7 anterior spinal fusion and that a screw in the C7 vertebral body protruded into the esophagus. The patient had been involved in an automobile accident in 2020 but reported no complications until approximately two years after the accident when dysphagia started. The CT scan did not reveal high-grade osseous stenosis of the spinal canal. A subsequent modified barium swallow test/fluoroscopic esophagogram confirmed that the screw caused the dysphagia.

Further consultation with the ear, nose, and throat (ENT), thoracic surgery, and neurosurgery departments led to a decision to remove the hardware. The patient underwent endoscopic surgery in the ENT department approximately three months after the initial CT scan. The neurosurgeon successfully removed the protruding screws using the Kocher maneuver. Given its structural integrity, the plate was fixed with adequate arthrodesis, and no other hardware was replaced. The patient experienced no postoperative complications, and the dysphagia resolved.

## Discussion

Although dysphagia has one of the highest complication rates at 5.3% following anterior cervical spine surgery, hardware failure leading to esophageal impingement is rare [5,6]. Screw migration into the esophagus can result in dysphagia and requires prompt intervention. Cases of esophageal perforation due to anterior cervical spine hardware placement have been previously reported. These complications are serious and require monitoring and immediate intervention.

In one study, clinical outcomes and revision rates were studied after ACDF on 28 patients followed over a minimum of one year. Within that population, the most common postoperative complication was transient dysphagia (32%). It was also reported that four (14%) of the patients required revision surgery at a median of 11.5 months postoperatively [7]. This is notable as it puts these patients at increased risk of undergoing another surgery.

In a different study, six patients who experienced pharyngoesophageal perforation following anterior cervical spine surgery were identified at an academic center [8]. All patients underwent repair with a sternocleidomastoid flap, with two requiring a further pectoralis myofascial flap for persistent esophageal leak. Ultimately, five patients were able to tolerate oral nutrition [8]. Our patient has also been able to tolerate oral nutrition and is closely monitored through a multidisciplinary approach, including gastroenterology, otolaryngology, and neurosurgery. Multidisciplinary collaboration is essential for the successful management of this condition, and early intervention can lead to more favorable outcomes. The limitations of this study include limited generalizability, considering that the evidence is based on one case, and the lack of long-term follow-up. Although the patient’s health is in good condition after the surgery, a long-term follow-up over 5-10 years postoperatively would be beneficial to identify any possible complications.

## Conclusions

This case highlights a rare yet significant complication of ACDF in which a migrated screw impinges on the esophagus, causing dysphagia. The patient's history of a motor vehicle accident and the subsequent delayed onset of symptoms underscores the importance of long-term monitoring in patients with spinal hardware. The successful resolution of dysphagia following the removal of the protruding screw by the ENT and neurosurgery teams demonstrates the effectiveness of prompt multidisciplinary intervention. This further emphasizes the need to consider hardware failure in the differential diagnosis of new-onset dysphagia in patients with a history of spinal surgery. Future studies should aim to improve our understanding of the mechanisms that lead to hardware migration and develop preventive strategies for such complications. Moreover, continued research on the management of dysphagia post-ACDF, particularly for identifying patients at an increased risk of hardware-related complications, is needed.
